# Correlates of socio-economic inequalities in women's television viewing: a study of intrapersonal, social and environmental mediators

**DOI:** 10.1186/1479-5868-9-3

**Published:** 2012-01-20

**Authors:** Megan Teychenne, Kylie Ball, Jo Salmon

**Affiliations:** 1 Centre for Physical Activity and Nutrition Research, School of Exercise and Nutrition Sciences, Deakin University, 221 Burwood Hwy, Burwood, VIC, 3125, Australia

**Keywords:** Television, socio-economic position, inequalities, women

## Abstract

**Introduction:**

Socio-economically disadvantaged women are at a greater risk of spending excess time engaged in television viewing, a behavior linked to several adverse health outcomes. However, the factors which explain socio-economic differences in television viewing are unknown. This study aimed to investigate the contribution of intrapersonal, social and environmental factors to mediating socio-economic (educational) inequalities in women's television viewing.

**Methods:**

Cross-sectional data were provided by 1,554 women (aged 18-65) who participated in the 'Socio-economic Status and Activity in Women study' of 2004. Based on an ecological framework, women self-reported their socio-economic position (highest education level), television viewing, as well as a number of potential intrapersonal (enjoyment of television viewing, preference for leisure-time sedentary behavior, depression, stress, weight status), social (social participation, interpersonal trust, social cohesion, social support for physical activity from friends and from family) and physical activity environmental factors (safety, aesthetics, distance to places of interest, and distance to physical activity facilities).

**Results:**

Multiple mediating analyses showed that two intrapersonal factors (enjoyment of television viewing and weight status) and two social factors (social cohesion and social support from friends for physical activity) partly explained the educational inequalities in women's television viewing. No physical activity environmental factors mediated educational variations in television viewing.

**Conclusions:**

Acknowledging the cross-sectional nature of this study, these findings suggest that health promotion interventions aimed at reducing educational inequalities in television viewing should focus on intrapersonal and social strategies, particularly providing enjoyable alternatives to television viewing, weight-loss/management information, increasing social cohesion in the neighborhood and promoting friend support for activity.

## Introduction

Sedentary behavior, defined as sitting behaviors that are performed at or slightly above the resting metabolic rate (1-1.5 METS) [1], has become increasingly prevalent in developed countries [2], with television viewing the most common leisure-time activity amongst adults from Australia [3], the UK [4] and the US [5]. Sedentary behaviors, particularly television viewing, have been linked to an increased risk of chronic health conditions independent of physical activity, including type 2 diabetes [6], obesity [7], cardiovascular disease [8] and metabolic syndrome [9]. Compared with their more advantaged peers, socio-economically disadvantaged adults (e.g. those with low education levels, low-status occupations or low incomes) are at a greater risk of spending excess time engaged in television viewing and screen-based entertainment [10]. Since these socio-economic differences in television viewing parallel socio-economic gradients in health outcomes [11,12], it is important to understand the mechanisms underlying the inverse relationship between socio-economic position and television viewing.

No previous studies have investigated mediators of socio-economic inequalities in television viewing among adults. Although research is limited, influences on adults' sedentary behavior, in particular television viewing, can be described in terms of intra-personal, social, and physical environmental factors, following the socio-ecological framework of human behavior [13]. Intra-personal influences on television viewing include weight status [14], energy intake [14], body dissatisfaction [15], and depressive symptoms [16]. Further, experiencing higher levels of stress is more prevalent amongst adults of a low socio-economic position [17], and stress has been reported as a barrier to physical activity amongst women [18] in physical activity literature, warranting research testing this possible mediator of the socio-economic gradient in television viewing. Social influences on television viewing include social support for physical activity [15]. Other social factors such as interpersonal trust (an indicator of perceived social capital) [19] and social cohesion (an absence of conflict within society) [20], have been found to predict physical activity in physical activity research. Yet, it is not known whether these factors predict television viewing, and moreover, whether these factors play a role in mediating the socio-economic gradient in television viewing. Very few studies have assessed physical environmental influences on television viewing. Nevertheless, one study suggested that the walkability of the neighborhood was inversely associated with television viewing in women [21].

Given that the extent to which these intrapersonal, social and physical environmental factors vary across socio-economic groups, and whether these variations explain socio-economic differences in television viewing, are unknown, the aim of this study was to examine the role of several intra-personal, social and physical environmental factors in mediating socio-economic (educational) inequalities in women's television viewing.

### Method

The present analyses were based on cross-sectional survey data collected from 1,554 women (aged 18-65) in 2004. Details of methods and recruitment are described in detail elsewhere [22,23] and summarised below.

#### Participants

Participants were recruited from 45 Melbourne suburbs with varying levels of socio-economic disadvantage, based on the Australian Bureau of Statistics SEIFA - Socio-economic Index for Areas [24]. Fifteen suburbs were randomly selected from each of the lowest, middle and highest socio-economic septiles, and the electoral roll was then used to randomly select women between the ages of 18 and 65 years living in those areas. Two separate samples of women were sent either a physical activity survey (n = 2,400) or a healthy eating survey (n = 2,400) and those who responded were given the opportunity to complete the alternative survey.

### Procedures

The study was approved by the Deakin University Human Research Committee. Surveys were posted out to the selected women. Following the Dilman protocol [25], non-respondents received a mailed reminder within three weeks and a second reminder with a replacement survey package a further three weeks later. Of those who were sent the physical activity survey, 1,045 women responded. Of those who were sent the healthy eating survey, 509 women also completed the physical activity survey. Of the resulting sample of 1554 women, a total of 14 women were excluded from analyses due to having recently moved out of the study neighborhoods. Additionally, a further 61 pregnant women were excluded. This left a total of 1,479 women whose data were included in the analyses.

### Measures

#### Predictor variable

Highest educational level was used as an indicator of individual socio-economic position. This was categorised as either 'no formal qualifications/up to year 10', 'year 12/trade/apprenticeship/certificate/diploma', or 'university degree/higher degree'. Education level has been argued as being a suitable proxy for socioeconomic position for women, since it overcomes issues relating to the instability of markers such as occupation and income that typically fluctuate for women who move in and out of the workforce during childrearing years [26].

#### Outcome variables

Participants were asked to estimate the number of hours and minutes they spent sitting watching television on a usual weekday, as well as a weekend day (in the past seven days). This measure has been found to have typical validity for similar self-report measures (r = 0.3, p < 0.01) [27] and has shown to be reliable (ICC = 0.82) in an Australian adult population [28].

#### Intra-personal Mediators

Five potential intra-personal mediators were assessed. Enjoyment of television viewing was assessed using a modified scale [29] (Cronbach's = 0.92; ICC = 0.83), which included 10 sets of opposing statements, each with a seven-point response scale, related to the feelings about television viewing (e.g. 1 "I enjoy it" to 7 "I hate it"). Preference for leisure-time sedentary behavior was assessed using a four-item measure with good reliability (ICC = 0.75) [28]. Participants were asked to indicate which type of activity (vigorous or moderate physical activity = 0; sedentary activity = 1) they would "most prefer" doing in various contexts (e.g. before work, during lunch breaks, after work, on the weekend). Mental health characteristics of participants were measured using the 30-item version of the General Health Questionnaire [30]. This includes questions relating to symptoms of depression, stress and anxiety experienced in the last couple of weeks as indicators of risk of poor mental health. The measurement properties of this tool have been widely reported and it has been found to provide an accurate prediction of those at risk of depression [31].

Level of stress was assessed using a four-item Perceived Stress Scale [32] (Cronbach's = 0.69; ICC = 0.64). Questions related to feelings of stress experienced in the last month. Participants reported on a five-point Likert scale (Never to Always) as to how often they felt such feelings (e.g. "How often is the last month have you felt that you were unable to control the important things in your life?"). Participants' weight status (Body Mass Index) was assessed through self-reported height and weight which has been shown to be valid in calculating body mass index in Australian women [33]. Scores on items assessing enjoyment of television viewing, preference for leisure-time sedentary behavior, depression, stress, and weight status responses were each summed and analyzed as continuous variables.

#### Social Mediators

Five potential social mediators were assessed in this study. Social participation was assessed using a 13-item measure adapted from Baum [34] (ICC = 0.73). Participants were asked to report on a four-point scale ranging from 'not at all' to 'more than twice a month' how frequently they participated in social activities (e.g. 'visited family or had family visit'). Interpersonal trust was assessed using two items which asked participants to rate on a 5-point Likert scale how strongly they agreed (1 = strongly disagree to 5 = strongly agree) with the statements "Most people can be trusted" and "Most of the time people try to be helpful" [35] (ICC = 0.75). Social cohesion within the community was assessed using a five-item measure which asked participants to rate on a 5-point Likert scale how strongly they agreed (1 = strongly disagree to 5 = strongly agree) with five statements (e.g. "People in the neighborhood can be trusted"; "People around here are willing to help their neighbours") [36] (ICC = 0.85). Social participation, interpersonal trust, and perceived social cohesion scores were each summed and analyzed as continuous variables.

Social support for physical activity was measured using two items adapted from published scales [37]. Participants were asked to report on a five-point scale ranging from 'never' to 'very often' (subsequently collapsed into three categories: never/rarely, sometimes, or often), how frequently they participated in physical activity with family (ICC = 0.96) and with friends/colleagues (ICC = 0.84) in the past year.

#### Physical environmental mediators

Four potential physical environmental mediators were assessed in this study. Perceived neighborhood aesthetics were measured with three items [38]. Participants were asked to indicate on a five-point Likert scale how strongly they agreed (1 = strongly disagree to 5 = strongly agree) with three statements (e.g. "My neighborhood is attractive"; "there are interesting walks to do"; (Cronbach's = 0.89; ICC = 0.90). Perceived safety in the neighborhood was assessed with three items (Cronbach's = 0.73; ICC = 0.80). Participants were asked to indicate on a five-point Likert scale how strongly they agreed (1 = strongly disagree to 5 = strongly agree) with three statements (e.g. "My neighborhood is safe for walking"; "the streets are well-lit at night") [38]. Perceived neighborhood aesthetics and safety scores were each summed and then analyzed as continuous variables.

Perceived distance to places of interest was assessed using a nine-item measure in which participants were asked to indicate whether a variety of places of interest (e.g. clothing shops, schools, pharmacies, playgrounds) were within walking distance from home (yes, no, don't know). Perceived distance to physical activity facilities (e.g. beach, golf course, gym/health centre, public open space, walking/bike paths) was assessed using a 10-item measure in which participants were asked to indicate whether a variety of places to be active were walking distance from home (yes, no, don't know). The number of 'yes' responses for both the perceived distance to places of interest (ICC = 0.97) and perceived distance to physical activity facilities (ICC = 0.83) were summed and analyzed as two continuous variables.

### Covariates

Marital status and children living at home were included in single and multiple mediating analyses as potentially confounding factors, since these were bivariately associated with television viewing (i.e. being married and having children living at home were associated with lower levels of television viewing). Other variables that were tested but found not to be associated with the outcome variable included long-term illness/injury, age, employment status and country of birth.

### Missing data

Cases were excluded where they did not contain complete independent or dependent variables for the specific analyses of interest. Variables with missing data included; Television viewing (1.2%), education (1.7%), enjoyment of television viewing (12%), stress (1.7%), preference for leisure-time sedentary behavior (2.5%), weight status (6.4%), depressive symptoms (7.9%), social cohesion (2.4%), interpersonal trust (2.1%), social participation (4.9%), family support (11.8%), friend support (9.4%), safety (3.2%), aesthetics (2.3%), distance to places of interest (2.9%), distance to physical activity facilities (39.2%), marital status (1.4%), children (0.0%).

### Statistical analyses

Analyses were performed using STATA version 11.0. Descriptive and unilevel analyses were used to examine the distributions of, and bivariate associations between, television viewing, demographic, education and mediator variables. MacKinnon's product of coefficients test of statistical mediation was used since it has been suggested that this method has greater statistical power than other commonly-used mediating methods [39]. The distributions of each variable were tested for normality and subsequently transformed to be as close as possible to a normal distribution using either a square root or log transformation. A total of five variables required a square root transformation (television viewing, depression, interpersonal trust, aesthetics and distance to places of interest) and two variables required a log transformation (weight status and social participation). A linear regression model (i.e. single mediating analysis) was used to bivariately estimate the contribution of intra-personal, social and physical environmental mediators to explaining educational variations in women's television viewing, controlling for clustering by neighborhood of residence. This was performed by following MacKinnon's product of coefficients formula (z = αβ/SEαβ), whereby α = the relationship between the independent variable (education) and the mediator, β = the relationship between the mediator and the dependant variable (television viewing), and SEαβ = the standard error of both α and β [39]. A z-score greater than the absolute value of 1.96 (i.e. greater than 1.96 or less than -1.96) was used to indicate a statistically significant mediating association. Following this, a multiple mediation analysis was performed, and only the proposed mediators that were found to be significantly associated with television viewing in single mediating analyses were included in the multiple mediation model.

## Results

Table 1 presents the socio-demographic characteristics of participants. The mean age of participants was 42 years (SD = 12.78) and just under a quarter of women (23%) reported not completing high school.

**Table 1:** Socio-demographic characteristics of participants (n = 1,479)

Characteristic	N	Percent
***Highest Qualification***		
Did not complete high school	329	23
High school/trade apprentice/Certificate diploma	595	41
University or Higher degree	539	37
***Age***		
Under 30 yrs	299	21
30 to 39 yrs	356	24
40 to 49 yrs	348	24
50+ yrs	454	31
***Country of birth***		
Australia	1097	74
UK	59	4
Italy	23	1
Greece	24	2
New Zealand	9	1
Vietnam	30	2
Other	235	16
***Marital Status***		
Married or defacto	937	64
Separated widowed or divorced	197	13
Never married	334	23
***Children living at home (up to 18 yrs)***		
Yes	601	40
No	888	60

***Employment status***		
Working full-time	559	38
Working part-time	359	25
Unemployed/laid off	31	2
Looking for work	25	2
Keeping house/raise children	254	17
Studying full-time	95	6
Retired	142	10
***Long term illness/injury***		
Yes	223	15
No	1252	85

The mean duration per week of time spent sitting watching television was just under 21 hours (Mean = 20.94, SD = 19.72). Education was inversely associated with women's television viewing (regression coefficient (τ) = -0.48; 95% CI = -0.62, -0.33). Table 2 presents the bivariable associations between education, and intra-personal, social and physical environmental factors hypothesised to mediate the relationship between education and television viewing. Of the intra-personal factors assessed, enjoyment of television viewing and weight status were significant mediators of educational variations in television viewing. Preference for sedentary behavior, stress and depressive symptoms were not found to be mediators of educational variations in television viewing.

**Table 2:** Potential mediators† from single mediating analyses explaining the association between education and television viewing amongst women

Potential mediators	α (95% CI)	β (95% CI)	αβ	SEαβ	z-score
***Intra-personal mediators***					
Enjoyment of television viewing	-1.76 (-2.77, -0.75)	0.03 (0.02, 0.04)	-0.057	0.02	-3.12*
Preference for sedentary behavior	-0.02 (-0.07, 0.02)	0.19 (0.03, 0.36)	-0.006	0.00	-1.24
Stress	-0.68 (-0.91, -0.45)	0.00 (-0.03, 0.03)	-0.000	0.01	-0.03
Weight status	-0.04 (-0.06, -0.03)	1.23 (0.79, 1.68)	-0.053	0.02	-3.43*
Depression	-0.08 (-0.19, 0.02)	-0.05 (-0.13, 0.02)	0.004	0.00	1.1
					
***Social mediators***					
Social cohesion	0.46 (0.24, 0.68)	-0.04 (-0.07, -0.01)	-0.018	0.01	-2.4*
Interpersonal trust	3.79 (2.49, 5.09)	-0.00 (-0.01, 0)	-0.019	0.01	-1.69
Social participation	0.06 (0.04, 0.07)	-0.51 (-1.01, -0.02)	-0.030	0.02	-1.98*
Social support from family	0.07 (0.01, 0.13)	-0.06 (-0.19, 0.06)	-0.005	0.01	-0.96
Social support from friends	0.11 (0.06, 0.17)	-0.30 (-0.42, -0.19)	-0.034	0.01	-3.1*
					
***Physical environmental mediators***					
Safety	0.20 (-0.03, 0.43)	-0.02 (-0.05, 0.02)	-0.003	0.00	-0.82
Aesthetics	13.96 (7.30, 20.62)	-0.00 (0, 0)	-0.03	0.02	-1.67
Distance to places of interest	1.20 (-0.32, 4.31)	-0.00 (-0.01, 0)	-0.003	0.00	-0.67
Distance to physical activity facilities	0.55 (0.33, 0.77)	0.04 (-0.02, 0.10)	0.024	0.02	1.36
					

*p < 0.05

† Adjusted for marital status and children and clustering by neighborhood

Social cohesion, social participation and social support from friends to be physically active were significant mediators of educational variations in television viewing. Interpersonal trust and social support from family to be physically active were not significant mediators of educational variations in television viewing. Furthermore, no physical environmental factors were found to be significant mediators of educational variations in television viewing.

Table 3 presents the results from multiple mediating analyses explaining the association between education and television viewing. This model included only those proposed mediators that were significantly associated with television viewing in single mediating analyses. Two intra-personal factors (enjoyment of television viewing and weight status) and two social factors (social cohesion and support from friends) remained significant mediators of educational variations in television viewing in the full model.

**Table 3:** Potential mediators† from multiple mediating analyses explaining the association between education and television viewing amongst women

Mediators	α (95% CI)	β (95% CI)	αβ	SEαβ	z-score
Enjoyment of television viewing	-1.76 (-2.77, 0.75)	0.03 (0.02, 0.04)	-0.058	0.02	-3.11*
Weight status	-0.04 (-0.06, -0.03)	-1.10 (0.58, 1.61)	-0.05	0.02	-3.05*
Social Cohesion	0.46 (0.24, 0.68)	-0.06 (-0.10, -0.02)	-0.028	0.01	-2.62*
Social participation	0.06 (0.04, 0.07)	-0.06 (-0.68, 0.55)	-0.004	0.02	-0.20
Social support from friends	0.11 (0.06, 0.17)	-0.25 (-0.39, -0.11)	-0.029	0.01	-2.63*

*p < 0.05

† Adjusted for marital status and children and clustering by neighborhood

## Discussion

To our knowledge, this is the first study to examine the role of intra-personal, social and physical environmental factors in explaining socio-economic differences in sedentary behavior (i.e. television viewing) amongst women. Consistent with previous literature, education was inversely associated with television viewing in women [40,41], emphasising the need to understand the underlying mechanisms that mediate this relationship. A major finding of this study was that the relationship between women's education level and television viewing was partly mediated by selected intra-personal and social factors, but not by physical environmental factors.

The current study suggested that less educated women were more likely to enjoy sedentary behavior in leisure-time, and this may explain their greater engagement in television viewing. Although the relationship between education and enjoyment of television viewing has not been assessed previously, similar trends have been found in the physical activity literature [23]. For example, one study found that the relationship between education and leisure-time walking was partly mediated by women's enjoyment of walking, suggesting that more educated women were more likely to enjoy and take part in leisure-time walking than less educated women [23]. In light of these findings, it may be that providing less educated women with enjoyable alternatives for relaxation during leisure-time (e.g. meditation, stretching, yoga) could be an important strategy to help reduce the higher levels of television viewing amongst socio-economically disadvantaged women.

The relationship between education and television viewing was also partly mediated by women's weight status. Consistent with a substantial body of research, it was found that less educated women were more likely to be overweight or obese than women with higher levels of education [11,42] and that being overweight was associated with greater television viewing in adults [14,43]. Therefore, interventions aimed at reducing the educational inequalities in television viewing may need to focus on weight-loss and weight-management approaches such as improving diet/healthy eating, overcoming body image concerns, as well as reducing television viewing and increasing physical activity amongst less educated women.

The current study provides support for the inverse association between social cohesion and television viewing [44-46], indicating that living in a more socially cohesive neighborhood was associated with spending less time watching television. Women with lower education living in areas with low perceived social cohesion may need further support to encourage them to make more active and less sedentary choices. Implementing walking groups, social support groups as well as other social activities that promote a cohesive neighborhood may be important strategies to increase social cohesion and thus contribute to reducing television viewing amongst less educated women.

Social support provided by friends for physical activity was found to partly mediate the relationship between education and women's television viewing. Consistent with previous research [47], women that indicated a high socio-economic position reported greater social support from friends for physical activity than did women who indicated a low socio-economic position. One possible explanation for this is that more educated women may be more likely to be employed in professional occupations which offer a wider social network in which women can draw on for social support [47]. The finding that greater social support for physical activity was inversely associated with television viewing may suggest that participating in physical activity with a friend displaces time spent watching television. Previous studies amongst women have indicated that television viewing often displaces physical activity [7,48], which may partly explain the association between television viewing and some health outcomes such as obesity [48]. Therefore, future interventions may need to focus on promoting social support for physical activity in order to reduce television viewing amongst less educated women.

Limitations of the current study include the cross-sectional design, which does not allow for causality or the direction of relationships to be determined. Self-report measures were used to assess television viewing as well as potential mediating factors. Although valid and reliable measures were used where possible, recall difficulties, error in judgment and socially desirable responses potentially limit the results. Further, the sample only included women and therefore it is not known as to whether the results of this study are generalizable to men. However, a major strength of this study is the consideration of multiple intra-personal, social and physical environmental mediators, which encompassed the constructs of the social ecological model [13]. Moreover, this study included a large sample of women from neighborhoods of varying levels of socio-economic disadvantage and therefore provided adequate power to detect associations, even after controlling for clustering by neighborhood key covariates. The use of a powerful multiple mediation analytical method was a further strength.

Since there is already a large pool of evidence relating to the relationship between education and physical activity, this study was novel as it focussed on the relationship between education and sedentary behaviour (i.e. television viewing), a group of behaviours recognised as being distinct from physical activity. The current study provided findings that suggest that focussing on intra-personal and social factors may be important in reducing the educational inequalities in women's television viewing. However, further studies including additional mediators are required to confirm these findings and to understand the reasons behind these educational differences. Providing enjoyable alternatives to television viewing, weight-loss/management information, increasing social cohesion in the neighborhood as well as friend support for activity amongst less educated women may be important strategies to reduce the educational inequalities in women's television viewing.

## Competing interests

The authors declare that they have no competing interests.

## Authors' contributions

MT performed the analyses and led the writing of the manuscript. KB conceived of the study, participated in the design, the survey development and helped to draft the manuscript. JS contributed to the survey questionnaire and helped to draft the manuscript. All authors read and approved the final manuscript.
